# The culture of olfactory ensheathing cells (OECs)—a distinct glial cell type

**DOI:** 10.1016/j.expneurol.2010.08.020

**Published:** 2011-05

**Authors:** Jennifer R. Higginson, Susan C. Barnett

**Affiliations:** Institute of Infection, Immunity and Inflammation College of Medical, Veterinary & Life Sciences (MVLS), Glasgow Biomedical Research Centre (GBRC), 120 University Place, Glasgow, G12 8TA, UK

**Keywords:** Olfactory ensheathing cells, Culture, Purification, Maintenance, Molecular and cellular, Properties

## Abstract

Olfactory ensheathing cells (OECs) have become a popular candidate for the transplant-mediated repair of the damaged CNS. In this review a description is made of the origins of these cells and a historical development of their purification and maintenance in culture. In addition, we illustrate the cellular and molecular characteristics of OECs and emphasise that although they share many properties with Schwann cells, they possess several inherent differences which may allow them to be more beneficial for CNS repair. In summary, OECs are distinct glial cells and the detailed understanding of their biological and molecular properties is essential in ensuring their clinical efficacy after cell transplantation. This article is part of a Special Issue entitled: Understanding olfactory ensheathing glia and their prospect for nervous system repair.

## Research Highlights

►Olfactory ensheathing cells are distinct glial cells. ►Purification, age and source of cells may influence their biological properties. ►Olfactory ensheathing cells possess properties that make them beneficial for CNS repair.

## Introduction

### Characteristics of OECs

The glial cells of the olfactory bulb were first identified by Golgi (1875) and Blanes (1898). Initially, they were considered to be Schwann cells of the olfactory system due to their location within the olfactory mucosa and olfactory bulb (Gasser, 1956; De Lorenzo, 1957) however, it soon became apparent that they were a distinct glial cell type. One of the first indications of their unusual properties came from an immunohistochemical study of olfactory nerves using antibodies to glial fibrillary acidic protein (GFAP), a marker generally considered to define astrocytes. These so-called olfactory nerve Schwann cells expressed GFAP leading to the suggestion that they also resembled astrocytes (Barber and Lindsay, 1982), a finding which was confirmed by others *in vitro* (Denis-Donini and Stenoz, 1988) and *in vivo* (Doucette, 1984). Subsequently three studies that followed reported that glial cells from the olfactory system expressed the low affinity NGF receptor/217c (Ran1), now known as p75^NTR^ (Pixley, 1992; Ramón-Cueto and Nieto-Sampedro, 1992; Barnett et al., 1993), typically a marker for non-myelin forming Schwann cells (Jessen et al., 1990). These studies shared a recurrent theme in that the cells were described as antigenically and morphologically very heterogeneous. In fact, variations in expression of GFAP led to the idea that olfactory glia comprised of both astrocyte-like cells and Schwann cell-like cells (Pixley, 1992; Franceschini and Barnett, 1996).

One of the complexities of understanding the biology of olfactory glial cells has been the variable methods used to purify them. In the study of Pixley (1992), a non-purified, mixed cell population was enzymatically dissociated from the olfactory mucosa of newborn rats and two types of cells were identified, termed Schwann cell-like olfactory nerve glial cells and astrocyte-like olfactory nerve glial cells. Both cell types expressed GFAP and S100β but the Schwann cell-like olfactory nerve glial cells resembled peripheral nerve Schwann cells due to their spindle-like morphology and p75^NTR^ expression. Astrocyte-like olfactory nerve glial cells, however, had a greater volume of cytoplasm around their nucleus with denser GFAP immunoreactivity and morphologically resembled astrocytes grown in serum-free media. These cells were also less abundant than Schwann cell-like olfactory nerve glial cells (Pixley, 1992). Schwann cell-like olfactory nerve glial cells have also been isolated from newborn rat olfactory bulbs (Chuah and Au, 1993). Similar astrocyte-like and Schwann cell-like cells were identified in post natal day 7 rat olfactory bulbs and polysialyated (PSA)-E-N-CAM (polysialylated (“embryonic”) form of neural cell adhesion molecule) was shown to be a marker for these astrocyte-like cells (Barnett et al., 1993; Franceschini and Barnett, 1996, Fig. 1). In this study, cells were purified using the oligodendrocyte 4 (O4) antibody and fluorescence activated cell sorting, but over time mainly p75^NTR^ positive spindle-shaped cells developed in culture (Franceschini and Barnett, 1996). Terminology from these early studies assigned olfactory bulb ensheathing cells (OBECs) to cells isolated from olfactory bulbs to distinguish them from olfactory nerve ensheathing cells (ONECs), however this classification is no longer used and the cells are now collectively referred to as olfactory ensheathing cells (OECs) or olfactory ensheathing glia (OEG); they will be referred to as OECs for the purpose of this review.

OECs have also been successfully cultured from adult (2.5 month old) rat olfactory bulb and were found to maintain both their ultrastructure and immunocytochemical properties shown *in vivo*, and their ability to ensheath neurites (Ramón-Cueto and Nieto-Sampedro, 1992, 1994; Ramón-Cueto et al., 1993). Their overall phenotype was reported to be 98.5% p75^NTR^ positive, multiform process bearing cells suggesting a tendency towards one type of olfactory glia (Ramón-Cueto et al., 1993). However, in a separate study, cells cultured from adult rat olfactory bulbs were found to display both astrocyte-like and Schwann cell-like morphologies. These subpopulations of cells displayed different migratory properties *in vitro* and could spontaneously transform from one type into another (Huang et al., 2008). It has been suggested that these Schwann cell-like OECs and astrocyte-like OECs may differ in other ways too. For example, it was observed that cultures from adult olfactory nerve rootlets had a lower proportion of PSA-(E)-N-CAM positive OECs when compared to cells isolated from the nerve fibre layer of the adult olfactory bulb (Kumar et al., 2005), which correlated with a lower ability to support dorsal root neurite outgrowth. This suggests that PSA-(E)-N-CAM positive astrocyte-like cells are less supportive of neurite outgrowth than Schwann cell-like OECs; though it was shown indirectly that these cells were still better than other glial and non-glial cell types (Kumar et al., 2005). However, these two OEC phenotypes have not been extensively studied by many other groups and therefore it is difficult to make firm conclusions about the role of such antigenic variants.

It is generally believed that OEC function *in vivo* is to ensheath olfactory receptor axons and to guide the regenerating axons back to the olfactory bulb during normal cell turnover, or after damage (Grazaidei and Monti-Graziadie, 1979, 1978; Doucette 1984; Raisman 1985). The olfactory receptor axons are non myelinated and they are organized in a similar manner to axons in an early stage of embryonic development in the peripheral nervous system i.e. axons which are grouped together within a single bundle wrapped by the glial cell cytoplasm. This “embryonic like” relationship remains unchanged throughout life. In contrast, although Schwann cells bundle many axons initially in the adult peripheral nerves, they usually ensheath non-myelinated axons with sheet like wrappings of cytoplasm which isolate axons from each other (Gasser, 1956; reviewed in Farbman, 1992). Interestingly, as the olfactory nerves develop, the number of axons ensheathed by OECs increase, whilst in other developing peripheral nerves the number of axons decrease (Fraher, 1982; Webster et al., 1973). Furthermore, the expression of PSA-(E)-N-CAM has been associated with areas of tissue involved in plasticity and cell reshaping under physiological conditions (Miragall et al., 1988; Caubit et al., 1993). Together, these data lead to the suggestion that OECs may remain in an embryonic state and studies have supported the view that OECs display morphological plasticity using time lapse (Van Den Pol and Santarelli, 2003) and after the addition of cAMP and endothelin-1 (Vincent et al., 2003).

Other studies have demonstrated differences in expression of markers between OECs generated from the nerve (present in the lamina propria, LP) and OECs generated from the olfactory bulb (OB) of neonatal mice (Au and Roskams, 2003). As OECs exit from the olfactory epithelium they display distinct and variable expression of p75^NTR^, S100β, GFAP, and labelling with the O4 antibody; characteristic markers of bulb OECs. LP-OECs also express a unique combination of developmentally important proteins, namely CD44, β1 integrin, P200, Notch 3, NG2, VEGF (Vascular endothelial growth factor), PACAP (Pituitary adenylate cyclase activating polypeptide) and CREB binding protein (CBP/p300), which have not been previously reported in olfactory bulb derived OECs (Au and Roskams, 2003). When a more detailed immunohistochemical study of the mouse olfactory bulb layer was made, differences were seen in expression of p75^NTR^, neuropeptide Y, GFAP and S100β, suggesting that different antigenic and morphological cells segregate this tissue into superficial and deep sublaminae (Au et al., 2002). It has also been reported that the glial cells in the olfactory bulb and mucosa are very similar in terms of proportions, morphology, rate of proliferation and expression of markers that change over time (Jani and Raisman, 2004; Richter et al., 2008). However, another study showed that there are fewer GFAP positive cells in the olfactory mucosa and that p75^NTR^ positive cells from the mucosa proliferate for longer than those from the olfactory bulb when placed in identical culture conditions (Au and Roskams, 2003). Proliferation of OECs appears to be variable in culture, this is quite apparent when comparing different studies, which could relate to the age of the donor animal (neonate versus adult), as well as the species from which the tissue is taken. This has been discussed in detail in two recent reviews (Kawaja et al., 2009; Tetzlaff et al., 2010).

## OECs and Schwann cells

**Fig. 2:**
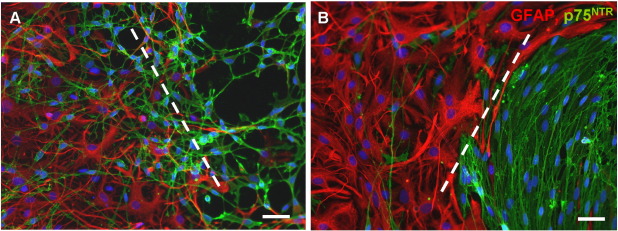
In confrontation assays, purified OECs mingle with astrocytes (A), however after treatment with Schwann cell conditioned media, a boundary is formed between the two cell type (B). The boundary is highlighted by a dotted line. OECs are labelled for p75^NTR^ (green) and astrocytes are labelled for GFAP (red). Scale bar = 20 μm.

It is true that OECs share many properties with Schwann cells and this has been discussed in many reviews (Ramón-Cueto and Valverde, 1994; Franklin and Barnett, 1997, 2000; Wewetzer et al., 2002; Franssen et al., 2007; Kocsis et al., 2009). However, whilst there is no doubt that there are striking similarities between these two cell types (Barnett et al., 2001; Thompson et al., 2000) there are also some very distinct differences, which were identified by looking at the way OECs and Schwann cells interact with astrocytes. From studies using cells purified from the neonatal olfactory bulb *in vitro* (Lakatos et al., 2000) and *in vivo* (Santos-Silva et al., 2007; Shields et al., 2000) it has been demonstrated that OECs, but not neonatal Schwann cells, can mingle with astrocytes and that the addition of Schwann cell conditioned media to OEC/astrocyte cultures causes boundary formation between the two cell types. The mechanism which regulates this difference is still unknown but several molecules appear to play a role including FGF2, N-cadherin, heparin sulphate proteoglycans and ephrins (Fairless et al., 2005; Santos-Silva et al., 2007; Afshari et al., 2010). From these studies it is apparent that Schwann cells secrete a factor(s) that prevent OECs from mingling with astrocytes, suggesting the secretory profile of each of these cell types is different (Fig. 2).

Interestingly, a study using cells purified from adult rats showed that a soluble factor secreted by meningeal cells affected Schwann cell clustering but not OEC cell clustering (Franssen et al., 2009), illustrating that these two cell types have differences in several biological properties and that these differences are seen in OECs isolated from neonatal or adult sources. It is likely that these distinct properties reflect the fact that OECs, unlike Schwann cells, naturally exist in a CNS environment and can coexist with astrocytes in the adult brain (Doucette, 1984, 1990; Raisman 1985).

## Generation and purification of OECs

Due to the ability of OECs to support continual outgrowth of olfactory receptor axons throughout life, their potential benefits in cell transplantation to repair the injured spinal cord have been the subject of a considerable amount of research over the past decade and have been discussed in other articles within this journal. It is clear that there is variability in the reparative potential of OECs and it is thought that this discrepancy depends on many factors. Differences in the efficiency of purifying OECs and the resultant proportion of contaminating cells in the culture are likely to be a factor, which will be discussed in more detail later. Moreover, the age, gender and species from which the cells are taken may also introduce variability, as will culture conditions, such as differences in media components and growth factors. In order to produce enough cells that will survive long enough *in vivo* to have some beneficial effect, cells must go through several passages prior to transplantation and the number of passages may also influence cell behaviour (Au et al., 2007).

The majority of research to date has been carried out using rodent olfactory tissue, but OECs have also been purified from dogs, pigs, primates and humans (Rubio et al., 2008; Techangamsuwan et al., 2008, 2009; Lim et al., 2010). A variety of different purification methods have been implemented and OECs can be cultured from both peripheral and central olfactory tissue. Interestingly it has been reported that primate OECs grow particularly well in serum containing medium for more than two months and do not senesce nor spontaneously immortalise (Rubio et al., 2008). Recent reviews by Kawaja et al., and Tetzlaff et al., provided an extensive critical analysis on the subject of OEC purification, the age and species on the donor tissue, and methods to culture cells which all may influence their biological properties; therefore we will only briefly discuss purification techniques here (Kawaja et al., 2009; Tetzlaff et al., 2010).

In some studies limited purification procedures have been carried out, for example the generation of cells from embryonic day 18 rat embryos by careful dissection of the olfactory nerve layer of the olfactory bulb (Doucette, 1993). This technique was based on the observation that OECs are the only cells present in this tissue during this period of development. Purification of adult OECs can also be achieved by exploiting the differential adhesion of OECs and other cells within the bulb such as fibroblasts and astrocytes. After enzymatic and mechanical dissociation of the outer olfactory nerve layer, cells were seeded onto uncoated plastic plates. The cells that remained in suspension after 1–2 h were found to be > 95% p75^NTR^ and S100β positive. This technique was modified slightly whereby cells were allowed to adhere for 48 h prior to the culture of cells in the supernatant and cultures were found to be 93.2% p75^NTR^ positive (Nash et al., 2001).

**Fig. 3:**
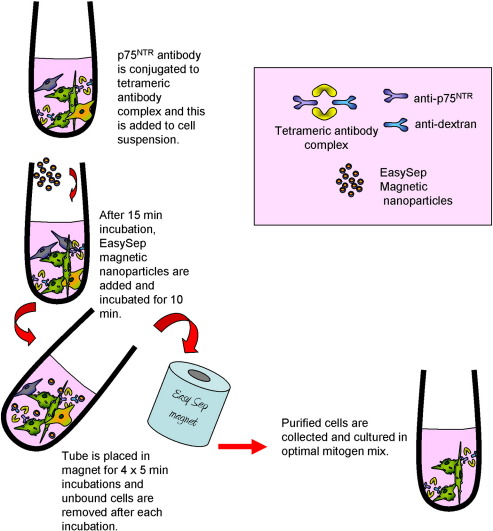
Summary of the method to purify OECs without FACS using the p75^NTR^ antibody and the EasySep™ kit from Stem Cell technologies. Purification is good and results obtained on transplantation and biology on interaction with astrocytes is the same, suggesting that the magnetic nanoparticles do not affect OEC function.

**Fig. 4:**
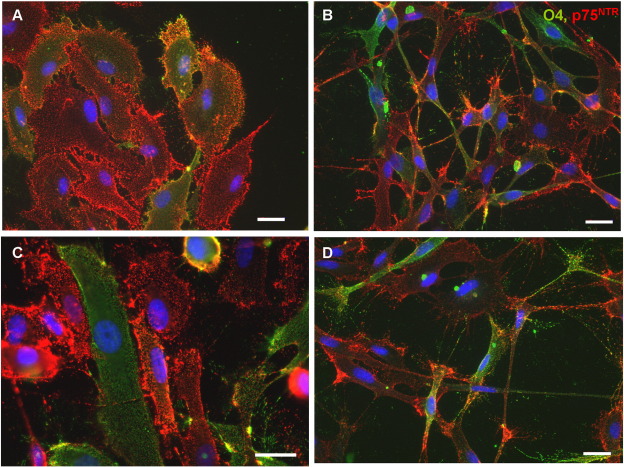
(A-D) The O4 antibody is continually expressed on purified OECs after passage and for many weeks. Purified OECs can possess a flat phenotype (A, C) and a more spindle phenotype (B, D). It has been suggested that the flatter OECs are entering senescence, however these cells continue to proliferate after passage in the optimal mitogen mix. Scale bar = 20 μm.

A different method was undertaken by Roskams et al., to purify OECs from neonatal mouse lamina propria, in which contaminating fibroblasts were removed with two rounds of Thy1.1 complement lysis (Au and Roskams, 2003). Another approach used to purify adult OECs is to immunopan using anti-p75^NTR^, since OECs express p75^NTR^ in culture (Ramón-Cueto and Nieto-Sampedro, 1994). This method positively selects for p75^NTR^ expressing cells by seeding them onto dishes coated with p75^NTR^ IgG. We have recently established a another system for purifying OECs from neonatal rat olfactory bulb by selecting for p75^NTR^ positive cells using magnetic nanoparticles and found that this generates a highly pure population of OECs (summarised in Fig. 3). OECs can also be purified from neonatal rat olfactory bulb by fluorescence activated cell sorting and using antibodies for both p75^NTR^ and the O4 antibody. Since the O4 antibody can label oligodendrocytes, another antibody, anti-galactocerebroside (GalC) is usually included in the sort in order to select for the O4 positive and GalC negative cell population, thus removing these O4 positive oligodendrocytes (Barnett et al., 1993). Furthermore, a study by Wewetzer et al., investigating the expression of the O4 antibody in unpurified cultured OECs from neonatal olfactory bulb concluded that, rather than being a glial specific marker, the O4 antibody was in fact expressed by olfactory receptor neurons and expression in OECs was due to the fact that OECs phagocytosed O4 positive axonal fragments (Wewetzer, et al., 2005). However, this does not explain the expression of O4 antibody on cells cultured *in vitro* for several weeks, following p75^NTR^ sorting (our observations, Fig. 4). A study carried out several years previously, showed that the introduction of FGF2 to the growth media enhanced expression of the O4 antibody in cultured OECs and that the marker was retained for several weeks (Alexander et al., 2002, Fig. 4). In the Wewetzer study, cells were cultured in DMEM containing 10% FCS, without any additional growth factors, which may explain their findings. Whilst their data convincingly shows phagocytosis of O4 positive axonal fragments, this could be an additional function of OECs *in vivo* but does it not eliminate the possibility that the O4 antibody is expressed endogenously.

It is clear, as highlighted by a recent review by Kawaja and colleagues, that many of the markers used to determine OEC purity (p75^NTR^, S100β and GFAP) are also expressed by Schwann cells (Kawaja et al., 2009). This author has suggested that smooth muscle actin and calponin are OEC specific (Boyd et al., 2006; Jahed et al., 2007) however, two independent reports cannot confirm this observation and contaminating non neural cells often express calponin and smooth muscle actin (Ibanez et al., 2007; Tomé et al., 2007; Toft et al., submitted). In fact, the main confirmation that OECs and Schwann cells are different comes from studying their interactions with astrocytes which has been discussed in the above sections. Interestingly, if the cells are purified using the O4 antibody or p75^NTR^ antisera by fluorescence activated cell sorting or with magnetic nanoparticles conjugated to anti-p75^NTR^ (Fig. 3) we have found that OECs still maintain the ability to mingle with astrocytes (Lakatos et al., 2000). This phenotypic difference strengthens the argument that current purification techniques can purify OECs effectively and may lead to the discovery of further genetic and phenotypic variation that will allow us to distinguish between OECs and Schwann cells. There has been some suggestion that current purification techniques could select for specific subtypes of OECs, when perhaps a more mixed population of cells would be more beneficial *in vivo*. However, this idea was dismissed when it was reported that OECs appeared to rapidly change through many phenotypes over time, thus confirming their morphological plasticity (Vincent et al., 2003; Van Den Pol and Santarelli, 2003).

In order for OECs to be a candidate for transplant-mediated repair, it is necessary to produce enough purified cells quickly prior to transplantation, however this may take several weeks following cell purification. It should be emphasised that if the purification of OECs from the olfactory mucosa or bulb is not carried out, a large number of other cell types will be isolated and propagated in culture, including fibroblast-like cells, mesenchymal stem cells, connective cells, immune cells and pericytes (Barnett and Chang, 2004; Tomé et al., 2009; Lindsay et al., 2010). The effect these contaminating cell types may exert on CNS repair after transplantation with OECs has not been systematically examined. It is therefore important to culture cells in optimal conditions which are conducive to both rapid and long-term proliferation as well as maintenance of cell-specific characteristics including morphology and antigenic properties. It has been shown that porcine OECs had a reduced capacity to remyelinate the rat spinal cord after injury the longer they were kept in culture (Radtke et al., 2010). The limited lifespan of primary cells in culture can be partially overcome by addition of growth factors or in the case of human cells, recent studies have used genetic manipulation to increase their proliferative capacity but also for the generation of a clonal cell line, a method which will be discussed later.

Initially serum-free conditioned media from type I astrocytes (astrocyte conditioned medium) (Noble and Murray, 1984) was found to be mitogenic for neonatal rat OECs but this proliferative capacity is short-lived (Alexander et al., 2002). Astrocyte conditioned medium was found to contain an isoform of neuregulin-1 (NRG-1), which is mitogenic and a survival factor for OECs (Pollock et al., 1999). Another source of neuregulin often used in OEC growth media is semipurified bovine pituitary extract, a crude source of GGF (glial growth factor) together with other growth factors e.g. FGF2 (De Mello et al., 2007). A systematic study was carried out to investigate the culture requirements of neonatal rat OECs and resulted in the idea that a combination of several mitogens was found to be optimal (Alexander et al., 2002; Yan et al., 2001). One such mixture of defined media, termed olfactory mitogen medium was devised that contained FGF2, forskolin, neuregulin beta 1 and 10 % astrocyte conditioned medium. It was found that culturing OECs in this defined medium allowed the cells to be maintained *in vitro* without losing their antigenic characteristics. Cells cultured in serum alone stop proliferating after 3–4 weeks in culture, whereas cells maintained in FGF2, forskolin, neuregulin beta 1 and 10 % astrocyte conditioned medium can proliferate for up to 9 weeks (Alexander et al., 2002). The molecular functions of the aforementioned different mitogens are partially understood through work which was carried out in Schwann cells. Forskolin upregulates expression of ErbB neuregulin receptors and elevates intracellular cAMP levels. The addition of forskolin has been shown to potentiate the mitogenic capacity of neuregulin and these mitogens act synergistically to enhance and prolong activation of the ERK/MAPK and Akt signalling pathways (Fregien et al., 2005; Monje et al., 2006; Rahmatullah et al., 1998). Whilst expression of p75^NTR^ remains constant in all culture conditions, with serum alone, cells have been shown to lose expression of glial markers such as the O4 antibody and PSA-(E)-N-CAM. Expression of the O4 antibody depends on FGF2 exposure and PSA-(E)-N-CAM expression is dependent upon specific culture conditions (Franceschini and Barnett, 1996). A separate study confirmed the finding that the combination of Hrg (a neuregulin beta-1 isoform) and FGF2 is mitogenic for adult OECs (Yan et al., 2001). The inconsistencies surrounding proliferative abilities of OEC culture therefore should be considered when comparing data from different studies.

Less is known about the correct culture conditions for maintaining OECs from species other than rat. Mouse OECs appear to have similar requirements to rat OECs with pituitary extract and forskolin added to the culture media (Au and Roskams, 2003; Richter et al., 2008), although we found that mouse OECs could not be maintained and expanded in culture in rat optimal mitogen media (unpublished observations). Canine OECs were found to respond to heregulin-1γ (Bock et al., 2007), whilst growth of porcine OECs in culture was found to be enhanced by forskolin. However, unlike cells cultured from other species, p75^NTR^ was down regulated after 4 weeks in culture in porcine OECs (Radtke et al., 2010). Adult primate OECs were successfully cultured for an extended period of time in media containing serum and did not require additional growth factors, whilst retaining OEC-specific markers (Rubio et al., 2008). This apparent contradiction of what we had previously learned from rodent studies highlights the complex nature of culturing OECs from different species.

## Purity of OECs for cell transplantation of CNS injury

If we are to use OECs for transplantation strategies in the clinic it will be necessary to grow large numbers of pure human cells from autologous sources. It was possible to grow human OECs from adult human olfactory bulb tissue although reports about their growth in culture are variable. In one study their response to growth factors was short-lived resulting in a reduced lifespan in culture (Barnett et al., 2000), but this is perhaps due to cell senescence as has been suggested for adult rat cells (Rubio et al., 2008). However, other studies have used human mucosal derived-OECs in rat models of SCI (Gorrie et al., 2010) and in clinical trials (Féron et al., 2005; Mackay-Sim et al., 2008) in which they describe good propagation of human cells using NT3 (Bianco et al., 2004). Recent work has been carried out on the transgenic expression of BMI1/TERT to generate an immortalised clonal human OEC cell line. The BMI1/TERT transgene can be excised after culture expansion, prior to transplantation in order to revert cells back to their former primary cell replicative state. This deimmortalisation step is necessary to eliminate adverse effects of uncontrolled proliferation. Reversible immortalisation of human OECs has been shown in young (13 years) and elderly donor tissue, which is encouraging for the future of autologous transplantation, however, deimmortalisation was not possible in all cultures raising questions about the safety of this procedure (García-Escudero et al., 2010; Lim et al., 2010). It has also been suggested that OECs should be isolated from olfactory mucosal biopsies since this method is a less invasive protocol for obtaining tissue. In fact cells prepared from mucosal biopsies were successfully grown up in large numbers for transplantation in a clinical trial (Féron et al., 2005). However, this is a very complex tissue and recent evidence suggests that mesenchymal stem cells as well as OECs could be isolated from this procedure (Tomé et al., 2009; Delorme et al., 2009; Lindsay et al., 2010).

There have been several reports suggesting that mixed populations of OECs and other cells, such as meningeal fibroblasts, are better for transplantation than purified OECs alone (Lakatos et al., 2003; Barnett and Chang 2004). Interestingly, it has been shown that OECs mingle with meningeal fibroblasts in culture whereas Schwann cells do not (Franssen et al., 2009). Furthermore, it has been reported that a 50:50 ratio of OECs with olfactory nerve fibroblast-like cells, rather than pure OECs produces optimal transplant-mediated repair (Li et al., 1998). However a recent study by Toft et al., has systematically analysed the effect of transplanting purified cell populations vs. mixed cell populations and found that purified OECs and Schwann cells induced less astrocyte reactivity following transplantation into a rat model of SCI when compared to cultures of mixed mucosa or combinations of fibroblasts with OECs or Schwann cells (Toft et al., submitted).

## Summary

OECs are distinct glial cells that have certain properties in common with Schwann cells but also possess other unique characteristics that may bestow advantages for CNS repair. Although it has been shown from rat models of SCI or even after transplantation into human patients that OECs can influence CNS repair albeit to a minor extent, it is clear that there are inconsistencies. Furthermore, it is also apparent that whilst OEC transplantation alone would not be sufficient to repair the damaged CNS, it may provide part of a more complex strategy. Thus, a thorough and extensive knowledge of the biological and molecular properties of OECs and the differences that can occur during their culture should be more closely assessed in order to ensure their clinical efficacy after cell transplantation.

## Figures and Tables

**Fig. 1:**
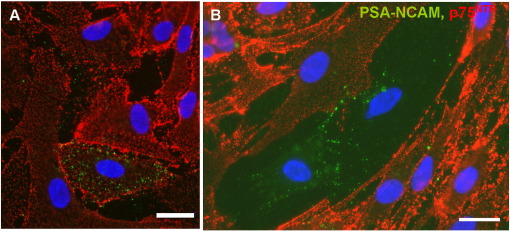
Purified OECs can be seen to express PSA-E-NCAM (green) together with p75^NTR^ (red, A) but also express PSA-E-NCAM alone (B). Scale bar = 20 μm.
